# Intestinal mantle cell lymphoma recurring at laparotomy scar region

**DOI:** 10.4103/0971-5851.65344

**Published:** 2009

**Authors:** Amitabh Ray, Ayan Basu, Jyotirup Goswami, Kalyan Bhattacharya

**Affiliations:** *Department of Radiation Oncology, AMRI Hospitals, India*

**Keywords:** *Incision line recurrence*, *intestinal NHL*, *mantle cell lymphoma*

## Abstract

Mantle cell lymphoma (MCL) is a moderately aggressive variety of non-Hodgkins lymphoma. Extranodal presentation of MCL is well known, the intestine being a common site. The incidence of colorectal involvement is relatively rare. Moreover, the recurrence of the disease at laparotomy scar site is even more rare. We report an unusual case of incision line recurrence in a case of colonic MCL occuring three years after initial treatment.

## INTRODUCTION

Mantle cell lymphoma (MCL) is a moderately aggressive variety of Non Hodgkins Lymphoma (NHL) accounting for about 7% of adult NHL.[1] A significant majority of cases present in Stage IV with the disease showing a predilection for occurrence in older males.[1] The gastro-intestinal tract is involved in approximately 20% of MCL cases.[1] Although incision scar recurrence is a known entity for other malignancies,[2] it is relatively unknown in NHL cases. We report one such case of scar recurrence in a case of MCL of the colon three years after initial treatment.

## CASE REPORT

A 62-year-old male gave a history of pain in left upper quadrant of abdomen for which he underwent investigation in 2005. He had suffered altered bowel movements, which had been progressively increasing over a period of three years. He was found to have a lump in left upper quadrant of abdomen with features suggestive of sub acute intestinal obstruction. There was no associated history of bleeding per-rectum. He did not have any B symptoms and CT scan of abdomen revealed a single large proliferative mass in the splenic flexure of the large gut. Colonoscopic biopsy was performed and was reported as lymphomatoid variant of Mantle cell lymphoma of the large gut. He subsequently underwent left hemicolectomy with ileo-colic anastomosis. Postoperative histopathology confirmed the colonoscopic biopsy report. Unfortunately, no immunohistochemistry was performed at this stage. His bone marrow study and staging workup were negative. He was thus classified as Stage IE with IPI score of 1. He subsequently underwent six cycles of chemotherapy using the CHOP (cyclophosphamide, doxorubicin, vincristine and prednisolone) regime. He had been on regular follow-up since then and had remained asymptomatic.

**Figure 1:**
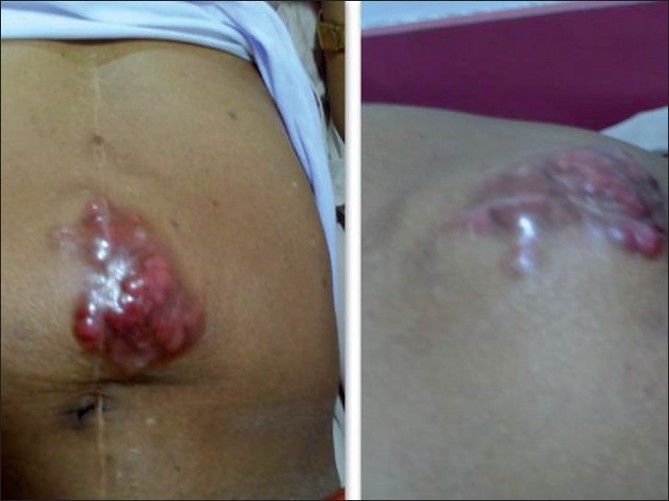
Scar recurrence at anterior abdominal wall

**Figure 2:**
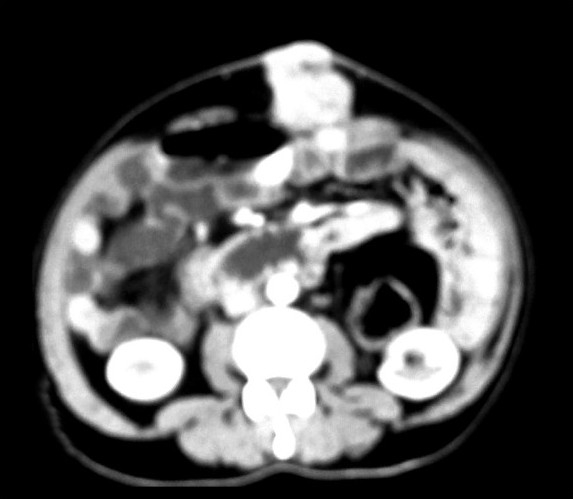
CT scan showing parietal mass in anterior abdominal wall

He had recurrence of abdominal pain in December 2008. This time he was found to have a mass in the supra umbilical region at the site of laparotomy incision scar [Figure 1]. He also had a complaint of painful bleeding piles. Clinical examination revealed left side axillary lymph node enlargement, a parietal anterior abdominal wall soft tissue mass and perianal nodules with bleeding piles. CT scan revealed a parietal mass in anterior abdominal wall [Figure 2]. He underwent biopsy from the abdominal mass and was found to have recurrent Non-hodgkins lymphoma. The histopathology was suggestive of a lymphoblastoid pattern. Immunohistochemistry was performed at this stage and was found to have Cyclin D1, CD5, CD19, CD20, and CD22 positivity. Bone marrow examination was positive for disease. He was thus diagnosed as having recurrent Non-hodgkin’s lymphoma of mantle cell variety with stage IV disease and was started on second line single agent Lenalidomide. He was found to be unresponsive to treatment and progressed even while on chemotherapy. He was subsequently taken up for palliative radiotherapy to the abdominal wall mass and perianal region, which yielded significant symptomatic relief. He is currently under follow-up and being considered for Bortezomib and rituximab based therapy.

## DISCUSSION

Primary gastro-intestinal (GI) tract lymphoma accounts for only 1 to 10% of all GI malignancies.[3] Nevertheless, the GI tract is the most common extra nodal site for lymphomas and accounts for 4-20% of all NHLs.[3] MCL was first distinguished by Weisenburger *et al*. It is a distinct clinicopathologic entity of NHL, characterized by a monotonous proliferation of small- to medium-sized lymphocytes with co-expression of CD5 and CD20 with CD10 and CD 23 negativity and moderately aggressive and incurable clinical course. MCL comprises 2.5-7% of all NHL, and the GI tract is involved in about 20% of cases.[4] The most frequent endoscopic finding of MCL is multiple lymphomatous polyposis (MLP).[1] This was, however, not the case for our patient who had a single large exophytic growth in the splenic flexure of the large gut.

The prognosis of GI MCL is guarded. Response to chemotherapy is seen in up to half of the patients.[5] COP (cyclophosphamide, doxorubicin, prednisolone), anthracycline-containing regimens, and CHOP (cyclophosphamide, doxorubicin, vincristine and prednisolone) have been used for MCL. Rituximab has also been used alone and in combination for MCL. Single agent rituximab has produced response rates of about 30%, and when combined with an anthracycline- containing regimen, response rates have increased to above 80%.[6] Bortezomib has been recently approved for treatment of recurrent MCL based on a multicenter trial with 155 patients, which demonstrated a response rate of 33% including 8% complete responses.[7] Lenalidomide is another novel agent showing promise in therapy of refractory mantle cell lymphomas.[8]

Radiotherapy has limited role in management of mantle cell lymphoma. Surgery too is of little use except in making tissue diagnosis. Chemotherapy remains the mainstay of treatment. The pattern of recurrence seen in this particular case might be a result of per-operative seeding of disease at anterior abdominal wall. This in itself is a very rare mode of disease recurrence and is rarer in gastrointestinal lymphoma cases. It is possible that avoidance of laparotomy might have prevented the morbidity of anterior abdominal wall recurrence in the first place. This case, hence, reiterates the importance of IHC in management of lymphomas and suggests the futility of major surgical intervention in mantle cell lymphomas.
